# Pre‐Extubation Low‐Dose Propofol in Pediatric Ear, Nose, and Throat Procedures: A Meta‐Analysis With Trial Sequential Analysis

**DOI:** 10.1002/pan.70277

**Published:** 2026-08-12

**Authors:** Rafaela Silva, Raphaela Mendes, João Mario Borges, Henrique Pivetta, Rafael Miranda, Isabela Alles, Vinicius Oliveira, Steven Staffa, Walid Alrayashi

**Affiliations:** ^1^ Department of Medicine Federal University of Maranhão São Luís Maranhão Brazil; ^2^ Department of Medicine Federal University of Minas Gerais Belo Horizonte Minas Gerais Brazil; ^3^ Department of Medicine Padre Albino University Center Catanduva São Paulo Brazil; ^4^ Department of Medicine University of South of Santa Catarina Pedra Branca Santa Catarina Brazil; ^5^ Department of Medicine University of Santa Cruz Do Sul Rio Grande do Sul Brazil; ^6^ Department of Medicine Federal University of Goiás Goiânia Goiás Brazil; ^7^ Department of Anesthesiology, Critical Care, and Pain Medicine Boston Children's Hospital and Harvard Medical School Boston Massachusetts USA

**Keywords:** Children, emergence delirium, extubation, general anesthesia, propofol

## Abstract

**Background:**

Post‐extubation emergence agitation (EA) is common in pediatric ear, nose, and throat (ENT) procedures and may lead to serious complications.

**Aims:**

To assess the efficacy of low‐dose propofol administered before extubation, compared with placebo, for preventing EA, using meta‐analysis with trial sequential analysis (TSA).

**Methods:**

We searched PubMed, Embase, and the Cochrane Central Register of Controlled Trials for randomized controlled trials (RCTs) comparing low‐dose propofol versus placebo administered before extubation in pediatric ENT procedures. The primary outcomes were laryngospasm, severity of EA, assessed by the Pediatric Anesthesia Emergence Delirium (PAED) scale score, and incidence of EA. Secondary outcomes included post‐anesthesia care unit (PACU) length of stay, time to recovery, and time to extubation. Random‐effects models were used to pool effect estimates with 95% confidence intervals (CIs). TSA was performed for all outcomes to assess the robustness of the evidence.

**Results:**

Ten RCTs were included in the review, of which six (414 pediatric patients) contributed to the meta‐analysis. Compared with control, propofol significantly reduced the incidence of EA (risk ratio [RR] = 0.57; 95% CI, 0.42–0.78) and lowered agitation severity (mean difference [MD] = −3.94; 95% CI, −5.74 to −2.13). Time to extubation was slightly longer (MD = 1.8 min; 95% CI, 1.4–2.3), whereas time to recovery (MD = 0.9 min; 95% CI, −1.9 to 3.6) and PACU length of stay (MD = −0.63 min; 95% CI, −3.2 to 1.9) did not differ. TSA confirmed the robustness of the findings for incidence of EA, severity of EA, and time to extubation. However, the certainty of evidence was very low for severity of EA and moderate for EA incidence and time to extubation. Laryngospasm outcomes were not pooled due to substantial heterogeneity.

**Conclusions:**

Our findings suggest that pre‐extubation propofol probably reduces the incidence of EA in pediatric ENT surgery, supported by moderate‐certainty evidence, and may lower EA severity measured by PAED scale scores, for which the certainty of evidence was very low. Propofol was also associated with a slightly longer time to extubation, with no difference in overall recovery time or PACU length of stay.

## 1 Introduction

Ensuring a smooth and safe emergence from general anesthesia remains one of the most critical challenges in pediatric surgical care [1, 2, 3]. Children undergoing ear, nose, and throat (ENT) procedures face an even higher risk during this phase due to surgical manipulation near the upper airway and the presence of accumulated secretions, both of which predispose them to laryngospasm [4]. At the same time, halogenated volatile anesthetics, which rank among the most commonly used agents in pediatric anesthesia, can activate neural pathways related to arousal and behavior, increasing the likelihood of emergence agitation (EA) [5, 6]. These are distinct, yet concurrent factors that make this period particularly challenging.

Incidence of EA has been reported in up to 80% of children receiving sevoflurane [7]. EA is characterized by a period of restlessness, agitation, inconsolable crying, disorientation, delusion, hallucination, and cognitive changes plus memory impairment. EA in children is generally short‐lived with no after‐effect; however, it is a troublesome phenomenon because it can result in injury to the patient or damage to the surgical site, lead to dissatisfaction and anxiety for the parents, and require extra nursing care with associated costs [8]. Additionally, in severe cases, laryngospasm can result in negative pressure pulmonary edema, which may prolong recovery in the post‐anesthesia care unit (PACU) or require increased respiratory support in intensive care units [9].

Low‐dose bolus administration of propofol given shortly before extubation following ENT surgery has been investigated as an adjunct to suppress airway reflexes and reduce emergence‐related agitation, due to its rapid onset, short duration, and antiemetic effects [10, 11, 12, 13, 14, 15, 16, 17, 18]. However, previous randomized controlled trials (RCTs) assessing pre‐extubation low‐dose propofol for preventing laryngospasm and EA have produced mixed results, and its role in routine pediatric practice remains unclear [10, 11]. To help clarify this gap in evidence, we performed a systematic review and meta‐analysis on the use of low‐dose propofol during the pre‐extubation period for reducing emergence‐related adverse events in children undergoing common ENT procedures.

## 2 Methods

Data registration, conduct of the meta‐analysis, and reporting adhered to the guidelines outlined in the *Cochrane Handbook for Systematic Reviews of Interventions* [19] and the *Preferred Reporting Items for Systematic Reviews and Meta‐Analyses* (PRISMA) statement [19, 20, 21]. The complete PRISMA checklist is available in Table S1. This systematic review and meta‐analysis was registered in the International Prospective Register of Systematic Reviews (PROSPERO; CRD420251056426) prior to study selection and data analysis. No new patient data were collected; therefore, institutional ethics approval was not required.

### 2.1 Eligibility Criteria

Studies were included if they met all of the following: (1) RCTs; (2) comparing low‐dose bolus administration of propofol (up to 1 mg·kg^−1^) versus placebo or no propofol during the pre‐extubation period; (3) conducted in pediatric patients undergoing ENT surgery; and (4) reporting at least one predefined outcome.

Articles were excluded if they (1) lacked a control group; (2) did not specify the timing of propofol administration; (3) were conference abstracts; or (4) involved overlapping study populations. No restrictions on language or date were applied.

### 2.2 Outcome Assessments

The primary outcomes were incidence of laryngospasm, severity of EA, and incidence of EA. Secondary outcomes included: (1) PACU length of stay, (2) time to recovery, and (3) time to extubation.

The severity of EA was assessed using the Pediatric Anesthesia Emergence Delirium (PAED) scale, a five‐item behavioral instrument that evaluates eye contact, purposeful actions, awareness of surroundings, restlessness, and consolability [22]. The PAED scale ranges from 0 to 20, with higher scores indicating greater severity of agitation.

Time to extubation recovery was defined as the time from cessation of anesthetic agents to eye opening or appropriate response to verbal commands. EA incidence was extracted as defined in the original trials. Outcomes were assessed during the immediate postoperative period. Data were pooled in meta‐analyses when at least three studies reported a given outcome.

### 2.3 Search Strategy and Data Extraction

A comprehensive search was conducted in MEDLINE (via PubMed), Embase, and CENTRAL between July and August 2025. No date or language restrictions were applied. The complete search strategy is provided in the Appendix S1. No restrictions on language or date were applied. Two reviewers (J.M. and R.M.) independently screened studies against eligibility criteria and extracted data using a predefined extraction form. Discrepancies were resolved by a third reviewer (R.S).

### 2.4 Quality Assessment

Two independent reviewers assessed the risk of bias in the individual trials and the methodological quality of the included studies. Discrepancies were resolved by a third reviewer (R.S.). To evaluate the risk of bias in RCTs, we used the Cochrane Risk of Bias 2 (RoB 2) tool [23]. For each domain, we assigned a judgment of “high risk”, “some concerns”, or “low risk” of bias.

Publication bias was assessed for every outcome with 10 or more studies, as inspection of funnel plots has limited value when the sample size is small, and the Egger test is only recommended when at least 10 studies are included [24]. The overall certainty of the evidence was evaluated using the Grading of Recommendations, Assessment, Development and Evaluation guidelines (GRADE) [25]. Studies were rated as providing very low, low, moderate, or high‐quality evidence based on the presence of risk of bias, inconsistency of results, imprecision, publication bias, and the magnitude of treatment effects.

### 2.5 Statistical Analysis

Mean differences (MDs) were used for continuous outcomes, and risk ratios (RRs) for dichotomous outcomes, estimated via the Mantel–Haenszel method. Pooled estimates were calculated using random‐effects models with REML estimators for τ^2^ and 95% confidence intervals (CIs), considering potential variations in clinical and methodological factors across studies. Statistical significance was set at *p* < 0.05, with emphasis on effect size magnitude and precision. Data synthesis required at least three studies per outcome, following Cochrane guidance. Analyses were conducted in RStudio (v4.5.0; RStudio, PBC) using the meta and metafor packages.

### 2.6 Assessment of Heterogeneity

Statistical heterogeneity was evaluated through visual inspection and quantified using the *I* ^2^ statistic and Cochran's *Q* test. Potential sources of heterogeneity were explored using leave‐one‐out analyses. When feasible, exploratory subgroup analyses were conducted to further investigate heterogeneity.

### 2.7 Trial Sequential Analysis

To determine whether the significant results of our meta‐analysis were reliable and conclusive, we conducted a trial sequential analysis (TSA, version 0.9.5.10 beta) using a random‐effects model for between‐study variance, a two‐sided *α* of 5% and *β* of 20% (80% power). This method estimates the required information size (RIS) comparable to a single adequately powered RCT and adjusts for heterogeneity and repeated testing. For these outcomes, results were considered robust if the cumulative Z‐curve crossed the conventional boundary, the trial sequential monitoring boundary, and the required information size, or entered the futility zone [26].

## 3 Results

### 3.1 Study Selection and Characteristics

Our systematic search yielded 1830 records. After duplicate removal and title and abstract screening, 12 records remained for full‐text review. Two RCTs were excluded at this stage: one because propofol was administered during induction rather than during the pre‐extubation period defined by our inclusion criteria [27], and another because the administered propofol regimen did not meet the eligibility criteria (Liao et al.). Consequently, 10 RCTs involving a total of 734 pediatric patients were included in the qualitative and quantitative synthesis. Figure 1 illustrates the PRISMA flow diagram of the study selection process.

**FIGURE 1:**
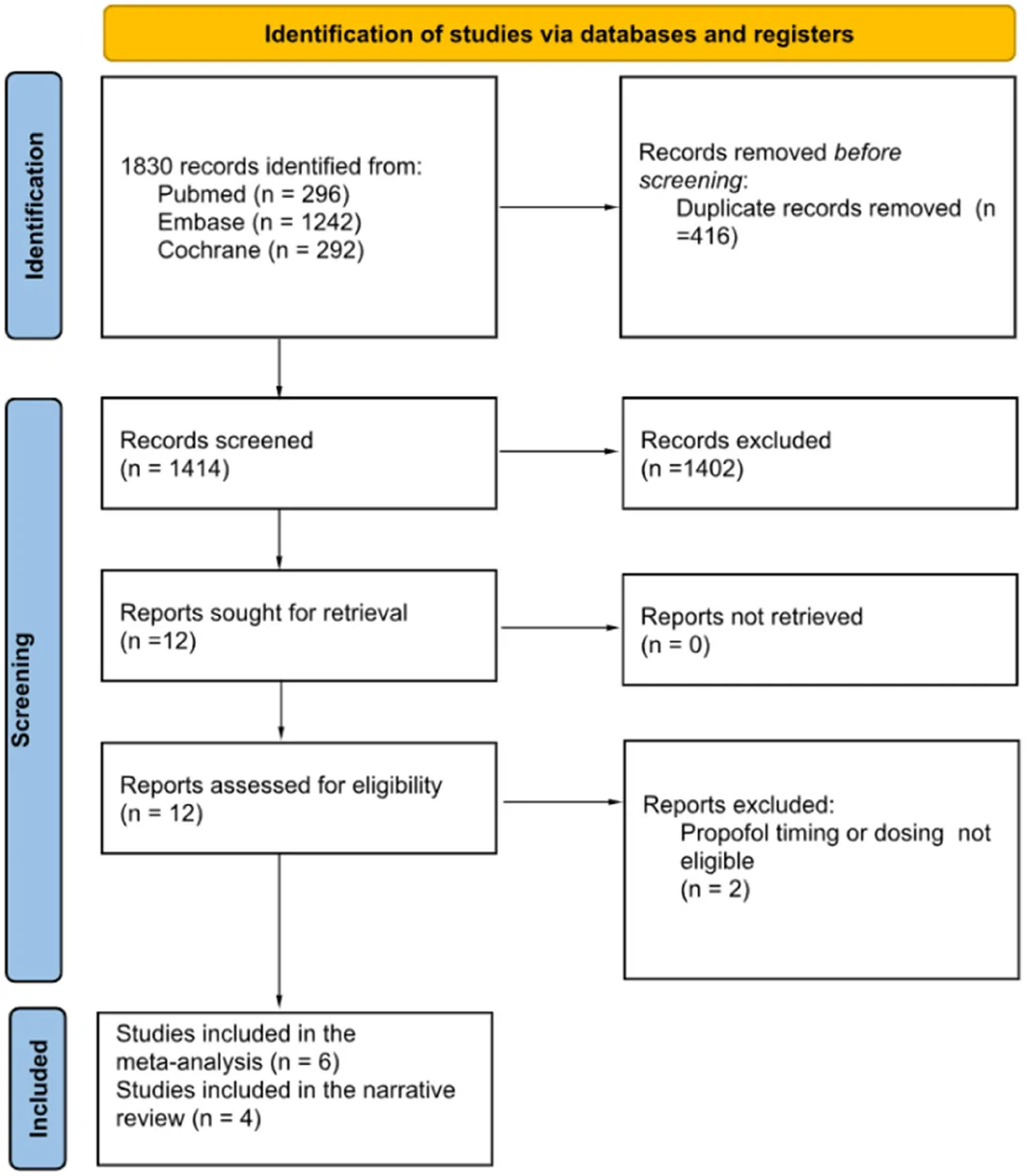
PRISMA 2020 flowchart.

Due to heterogeneous outcome definitions, four studies contributed only to the narrative synthesis of laryngospasm. The meta‐analysis included six RCTs involving 414 patients, of whom 207 (50%) were randomized to receive propofol and the remaining 207 (50%) were randomized to receive saline placebo. In all included trials, propofol was administered as a single intravenous dose of 1 1 mg·kg^−1^.

The mean age across studies ranged from 3.9 to 9.6 years, and the mean weight ranged from 18.5 to 29.5 kg. Four studies included only ASA class 1 patients [11, 13, 14, 18]; two included ASA class 2 patients [12, 16]. Four studies did not report ASA physical status [15, 17, 28, 29].

Five studies included patients undergoing adenotonsillectomy [11, 13, 15, 16]. Five studies included tonsillectomy procedures, and one also included adenoidectomy cases. Eight studies administered a propofol dose of 1 mg·kg^−1^, while two administered a dose of 0.5 mg·kg^−1^. Administration timing varied across studies, ranging from 10 min before the end of surgery to its exact completion.

Sevoflurane was the most commonly used anesthetic agent, serving as the induction agent in five studies and the maintenance agent in five. Thiopental was used for induction in two studies, whereas isoflurane was used for maintenance in four. For neuromuscular blockade, atracurium was the most frequently used agent (six studies), while rocuronium, cisatracurium, vecuronium, and suxamethonium were each used in one study. Postoperative analgesic strategies varied across studies: two used paracetamol, two used nonsteroidal anti‐inflammatory drugs (NSAIDs), and the remaining studies did not report postoperative analgesia. Additional study and patient characteristics are summarized in Table 1.

**TABLE 1:** Baseline characteristics of included studies.

Study	Sample size Propofol Control	Surgery	Control group	Propofol Dose (mg/kg)	Timing of propofol	Induction agents and doses	Maintenance of anesthesia	Postoperative analgesia	Age, y Mean (SD) Propofol Control	Weight, kg Mean (SD) Propofol Control	Male *n* (%) Propofol Control	ASA class, %	Surgery duration, min Mean (SD) Propofol Control	Outcomes
Abdellatif & Ali 2013 [15]	40 40	T&A	Placebo	1	5 min before the end of the surgery	8% sevoflurane in 70% N_2_O/O_2_ + rocuronium 0.6 mg/kg IV	60% N_2_O/O_2_ + sevoflurane 2%–3% end‐tidal	Paracetamol 15 mg/kg IV + dexamethasone (max 16 mg)	4.2 (1.4) 3.9 (1.6)	19.8 (4.6) 18.7 (4.5)	24 (60) 22 (55)	NA	38.6 (12.2) 35 (10)	Laryngospasm, severity of EA, incidence of EA
Bakhamees 2009 [13]	40 40	T&A with or without BMT	Placebo	1	End of the surgery	7% sevoflurane in 50% O_2_/N_2_O + atracurium 0.5 mg/kg IV	Age‐corrected MAC of sevoflurane in 50% O_2_/N_2_O	Rectal paracetamol 40 mg/kg	4.8 (1.9) 5.1 (1.9)	22.3 (3.1) 21.3 (3.3)	17 (42.5) 15 (37.5)	I: 100 II: 0 III: 0	43.3 (6.6) 45.3 (7)	Incidence of EA
Batra 2005 [12]	60 60	Tons with or without AD	Placebo	0.5	1 min before extubation	O_2_ + N_2_O + sevoflurane + Suxamethonium 1.5 mg/kg IV	Isoflurane 1.5%–2.5% in 66% N_2_O/O_2_	NA	5.6 (2.5) 6.1 (2.7)	24.5 (8) 26.7 (9)	38 (63.3) 36 (60)	I: 78.3 II: 21.7 III: 0	33.5 (6.4) 29.7 (6)	Laryngospasm (narrative only)
Hosseini 2022 [14]	35 35	Tons	Placebo	0.5	End of the surgery, before extubation	Propofol 2.5 mg/kg IV + atracurium 0.5 mg/kg IV	Isoflurane 1% + equal mixture of N_2_O/O_2_	NA	6.66 (2.6) 7.17 (2.8)	24.83 (11.14) 26.11 (1.7)	17 (48.6) 19 (54.3)	I: 100 II: 0 III: 0	NA	Laryngospasm (narrative only)
Iqbal 2017 [28]	40 40	Tons	Standard care	1	Before extubation	Propofol 2 mg/kg IV + atracurium 0.5 mg/kg IV	50% O_2_/N_2_O + isoflurane 1%–1.5%	NA	9.60 (2.85) 9.45 (2.6)	20.33 (7.45) 19.53 (7.82)	18 (45) 17 (42.5)	NA	NA	Laryngospasm (narrative only)
Kaur 2006 [29]	25 25	T&A	Standard care	1	Before suction	Propofol 2 mg/kg IV + vecuronium 0.1 mg/kg IV	O_2_ 40% + N_2_O 60% + halothane 0.75%–1%	NA	8.70 (3.16) 8.96 (2.79)	28.90 (7.6) 29.36 (6.5)	22 (88) 18 (72)	NA	NA	Laryngospasm (narrative only)
Lee 2010 [11]	44 44	T&A without My	Placebo	1	End of the surgery	Thiopental sodium 5 mg/kg IV + atracurium 0.5 mg/kg IV	O_2_ 2 L/min + N_2_O 2 L/min + sevoflurane 2–2.5 vol%	Ketorolac 1 mg/kg IV 20 min before end of surgery	5.8 (1.9) 6.3 (1.6)	22.4 (7.2) 24.2 (6.4)	26 (59.1) 25 (56.8)	I: 100 II: 0 III: 0	37.7 (12.4) 34.5 (9)	Laryngospasm, severity of EA, incidence of EA
Mahdavi Rad 2019 [17]	30 30	Tons	Placebo	1	After discontinuation of isoflurane	Thiopental 6 mg/kg + atracurium 0.5 mg/kg	Isoflurane	NA	7.60 (2.36) 6.86 (2.7)	28.70 (14.64) 28.07 (31.46)	20 (66.7) 22 (73.3)	NA	NA	Severity of EA and incidence of EA
Rizk & Samir 2014 [16]	30 30	T&A or Ad	Placebo	1	10 min before the end of surgery	5%–8% sevoflurane in O_2_ + atracurium 0.5 mg/kg IV	Sevoflurane	Diclofenac suppository 1–2 mg/kg	4.7 (1.5) 4.1 (1.3)	26.3 (3.2) 24.9 (4.7)	16 (53.3) 13 (43.3)	I: 88.3 II: 11.7 III: 0	NA	Severity of EA and incidence of EA
Zhong 2018 [18]	23 23	Tons	Placebo	1	End of the surgery	O_2_ air mixture with sevoflurane + cisatracurium 0.2 mg/kg IV	Sevoflurane	NA	4.65 (0.88) 4.52 (1)	18.69 (2.8) 18.52 (2.76)	14 (60.9) 14 (60.9)	I: 100 II: 0 III: 0	31.82 ± 3.55 30.65 ± 3.02	Severity of EA and incidence of EA

Abbreviations: Ad, adenoidectomy; ASA, American Society of Anesthesiologists physical status classification; BMT, bilateral myringotomy; My, myringotomy; NA, not available; PPF, Propofol; SD, standard deviation; T&A, adenotonsillectomy; Tons, tonsillectomy.

### 3.2 Emergence‐Related Adverse Events

Propofol significantly reduced the severity of EA, lowering PAED scale score across five trials [11, 15, 16, 17, 18] including 335 patients (MD = −3.94; 95% CI, −5.74 to −2.13; *p* < 0.001; *I* ^2^ = 81.6%; Figure 2A).

**FIGURE 2:**
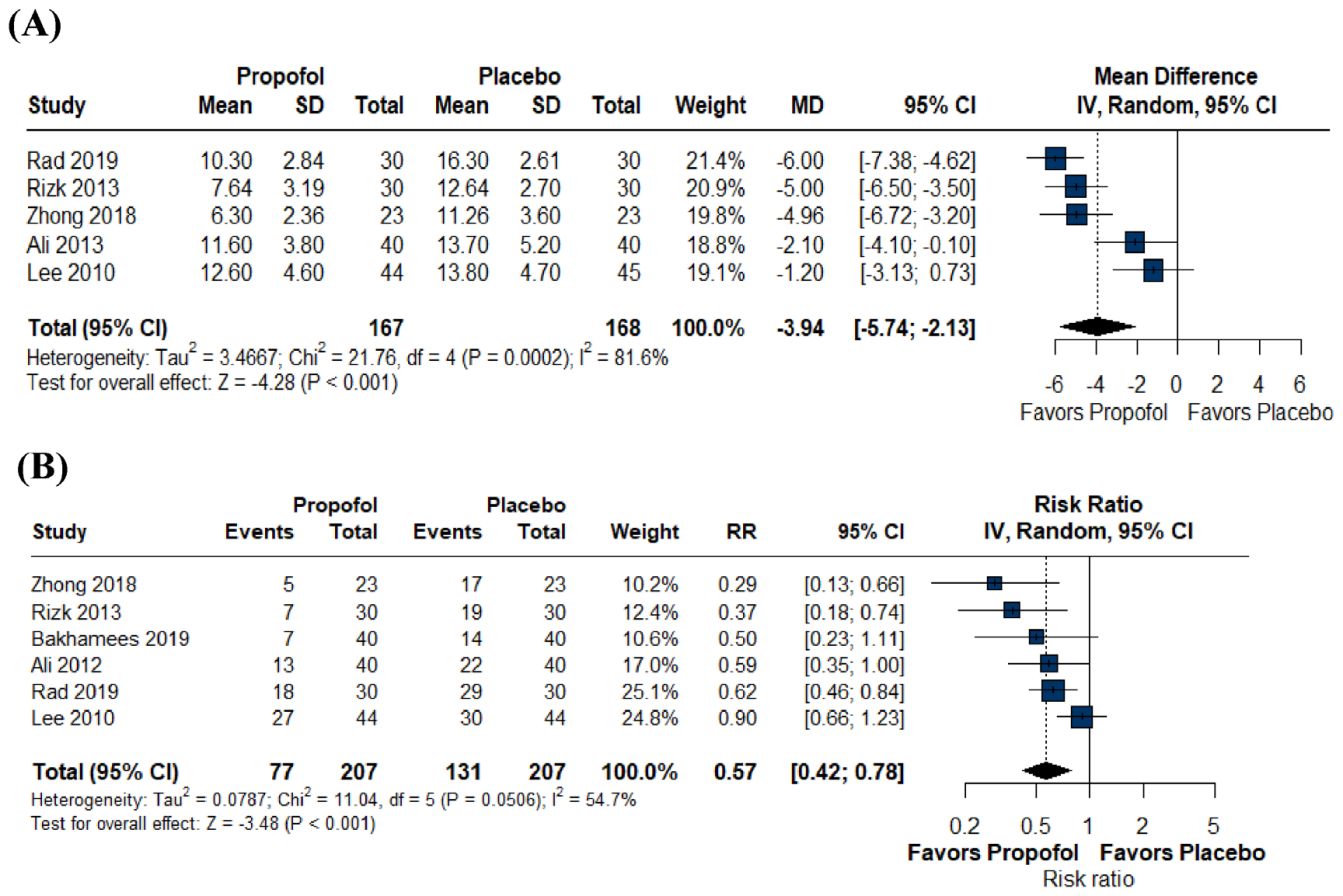
Effect of low‐dose propofol on emergence‐related adverse events. (A) Severity of emergence agitation, as measured by the Pediatric Anesthesia Emergence Delirium (PAED) scale score. (B) Incidence of emergence agitation. CI, confidence interval; MD, mean difference; RR, risk ratio; SD, standard deviation.

In addition, propofol was also associated with a lower risk of EA, based on six studies [11, 13, 15, 16, 17, 18] with 414 patients (RR = 0.57; 95% CI, 0.42–0.78; *p* < 0.001; *I* ^2^ = 54.7%; Figure 2B).

Laryngospasm was reported in six trials [11, 12, 14, 15, 28, 29] but was assessed under heterogeneous clinical conditions. Definitions of laryngospasm were inconsistent across studies. Reported event rates were low overall. Among studies using non‐sevoflurane maintenance, one trial reported fewer laryngospasm events with propofol (1/40 vs. 17/40), whereas the remaining three found no significant between‐group differences. Neither of the sevoflurane‐based studies reported laryngospasm events in either group. Owing to substantial heterogeneity in anesthetic techniques, extubation conditions, and outcome definitions, quantitative synthesis was not performed.

No instances of apnea or other expected adverse effects related to propofol administration during emergence were reported in any of the included trials.

### 3.3 Anesthetic Recovery Profile

Time to extubation was prolonged in patients receiving propofol compared with the control group in four trials [11, 15, 17, 18] including 274 patients (MD = 1.8 min; 95% CI, 1.4–2.3; *p* < 0.001; *I* ^2^ = 62.5%; Figure 3A).

**FIGURE 3:**
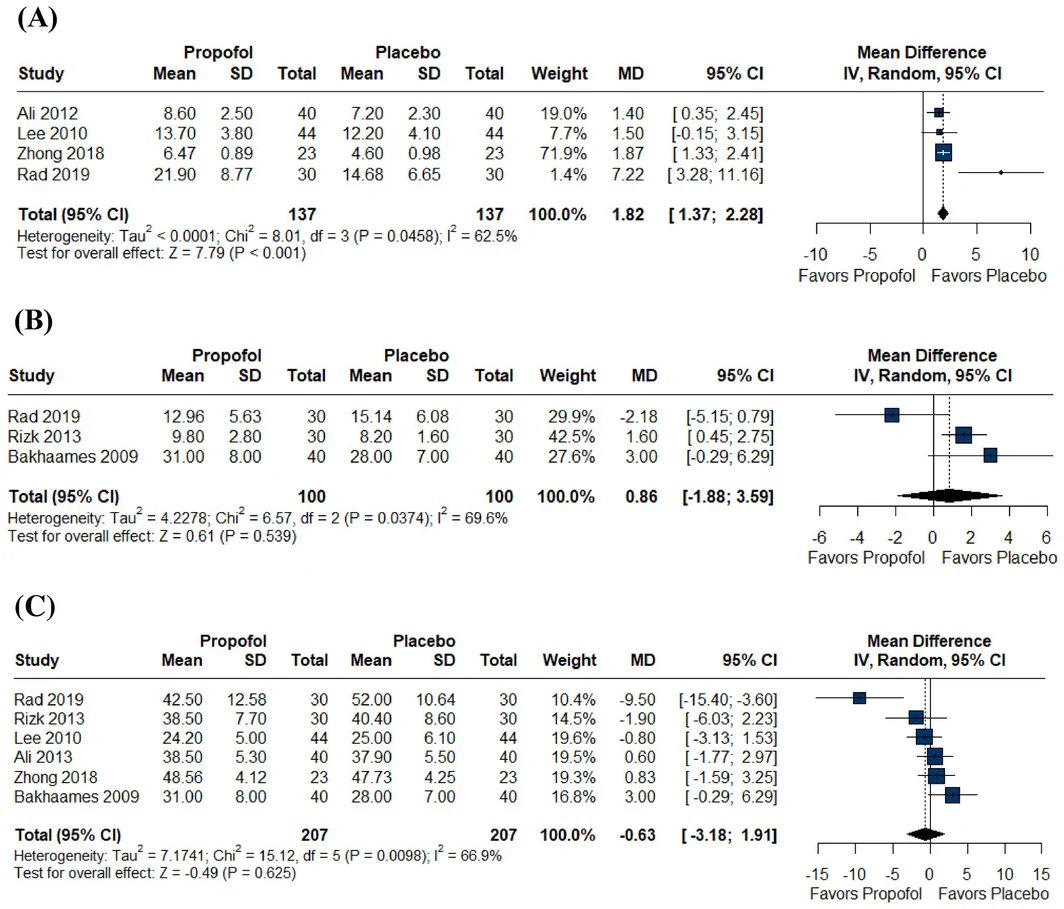
Effect of low‐dose propofol on anesthetic recovery profile. (A) Time to extubation. (B) Time to recovery. (C) Duration of stay in the post‐anesthesia care unit. CI, confidence interval; MD, mean difference; RR, risk ratio; SD, standard deviation.

Three trials [13, 16, 17] involving 200 patients reported time to recovery, which showed no statistically significant difference between low‐dose propofol and control groups (MD = 0.9 min; 95% CI, −1.9 to 3.6; *p* = 0.539; *I* ^2^ = 69.6%; Figure 3B) [2]. Similarly, no significant difference was observed in PACU length of stay across 414 patients in six trials [11, 13, 15, 16, 17, 18] (MD = −0.63 min; 95% CI, −3.2 to 1.9; *p* = 0.62; *I* ^2^ = 67%; Figure 3C).

### 3.4 Subgroup Analyses

#### 3.4.1 Timing of Propofol Bolus Administration

Exploratory subgroup analyses stratified by the timing of propofol bolus administration were performed for EA outcomes to explore potential sources of heterogeneity. The results are presented in Figure S1. For EA severity assessed using the PAED scale, a significant reduction favoring propofol was maintained in the pooled “administration at surgical completion” subgroup (3 trials: [11, 17, 18] MD = −4.11; 95% CI, −6.94 to −1.28; *p* = 0.004; *I* ^2^ = 87.5%; Figure S1A), as well as in the single trials in which propofol was administered 10 min [16] and 5 min [15] before surgical completion.

Similarly, the incidence of EA was significantly reduced across all timing strata, including the pooled “administration at surgical completion” subgroup (4 trials: RR = 0.60; 95% CI, 0.40 to 0.91; *p* = 0.02; *I* ^2^ = 63.3%; Figure S1B), the single trial in which propofol was administered 10 min before completion, and the single trial in which it was administered 5 min before completion.

#### 3.4.2 Maintenance Agent Subgroup

Exploratory subgroup analyses were conducted by stratifying trials into sevoflurane and non‐sevoflurane cohorts. The results are presented in Figure S2. Because the non‐sevoflurane stratum comprised only a single trial (Mahdavi Rad [17]), this stratification effectively functioned as a leave‐one‐out sensitivity analysis to assess the robustness of our overall estimates and systematically identify sources of clinical heterogeneity.

Regarding EA severity assessed using the PAED score, the beneficial effect of propofol remained significant within the pooled sevoflurane subgroup (4 trials: [11, 15, 16, 18] MD = −3.38; 95% CI, −5.30 to −1.46; *p* = 0.0005; Figure S2A), while high statistical heterogeneity persisted (*I* ^2^ = 78.2%). Similarly, the incidence of EA remained significantly reduced with propofol within the pooled sevoflurane cohort (5 trials: [11, 13, 15, 16, 18] RR = 0.54; 95% CI, 0.35 to 0.82; *p* = 0.004; Figure S2B), with heterogeneity remaining largely unaffected (*I* ^2^ = 63.4%).

#### 3.4.3 PAED Assessment Subgroup

Exploratory subgroup analyses were conducted according to the method and timing of PAED assessment used to evaluate EA severity. The results are presented in Figure S3. Most studies assessed PAED scores at PACU arrival [15, 16, 17], one study assessed patients 5 min after PACU arrival [11], and another performed serial assessments every 5 min throughout PACU stay, reporting the highest PAED score recorded for each patient [18]. Propofol was associated with significantly lower PAED scores in studies assessing patients at PACU arrival (3 trials: MD = −4.46; 95% CI, −6.68 to −2.25; Figure S3) and in the study using continuous assessment (MD = −4.96; 95% CI, −6.72 to −3.20; Figure S3), whereas no significant difference was observed in the study evaluating patients 5 min after PACU arrival (MD = −1.20; 95% CI, −3.13 to 0.73; Figure S3). The significant test for subgroup differences (*p* = 0.0122) suggests that variations in the timing and methodology of PAED assessment may have contributed to the substantial heterogeneity observed in the overall analysis.

#### 3.4.4 Emergence Agitation Cutoff Subgroup

Exploratory subgroup analyses were also performed according to the cutoff used to define EA. Most studies employed Aono's 4‐point scale using a threshold of ≥ 3 [11, 15, 16, 18], whereas one study [13] defined EA as a score ≥ 5 on a clinical 10‐point scale and another used a PAED score ≥ 10 [17]. Within the Aono subgroup, propofol remained associated with a significantly lower incidence of EA (4 trials: RR = 0.54; 95% CI, 0.32–0.89; *p* = 0.015; Figure S4), closely mirroring the overall pooled estimate (RR = 0.57; 95% CI, 0.42–0.78; *p* < 0.001; Figure 2). Moreover, the absence of significant subgroup differences (*p* = 0.81) suggests that variations in the cutoff used to define EA were unlikely to have materially influenced the overall findings or contributed substantially to between‐study heterogeneity.

### 3.5 Trial Sequential Analysis

Trial sequential analysis (Figure 4) supported the robustness of the findings across the analyzed outcomes, as the cumulative sample size exceeded the RIS thresholds in all analyses. For both the severity (RIS = 117; Figure 4A) and incidence (RIS = 359; Figure 4B) of EA, the cumulative Z‐curve crossed the conventional significance boundaries, additionally crossing the sequential monitoring boundary for benefit in the incidence analysis. Conversely, for time to extubation (RIS = 187; Figure 4C), the cumulative Z‐curve crossed the sequential monitoring boundary for harm, favoring the control group. Overall, exceeding the RIS thresholds suggests that the available evidence is sufficiently powered for these outcomes and reduces the likelihood that the observed findings are attributable to random errors.

**FIGURE 4:**
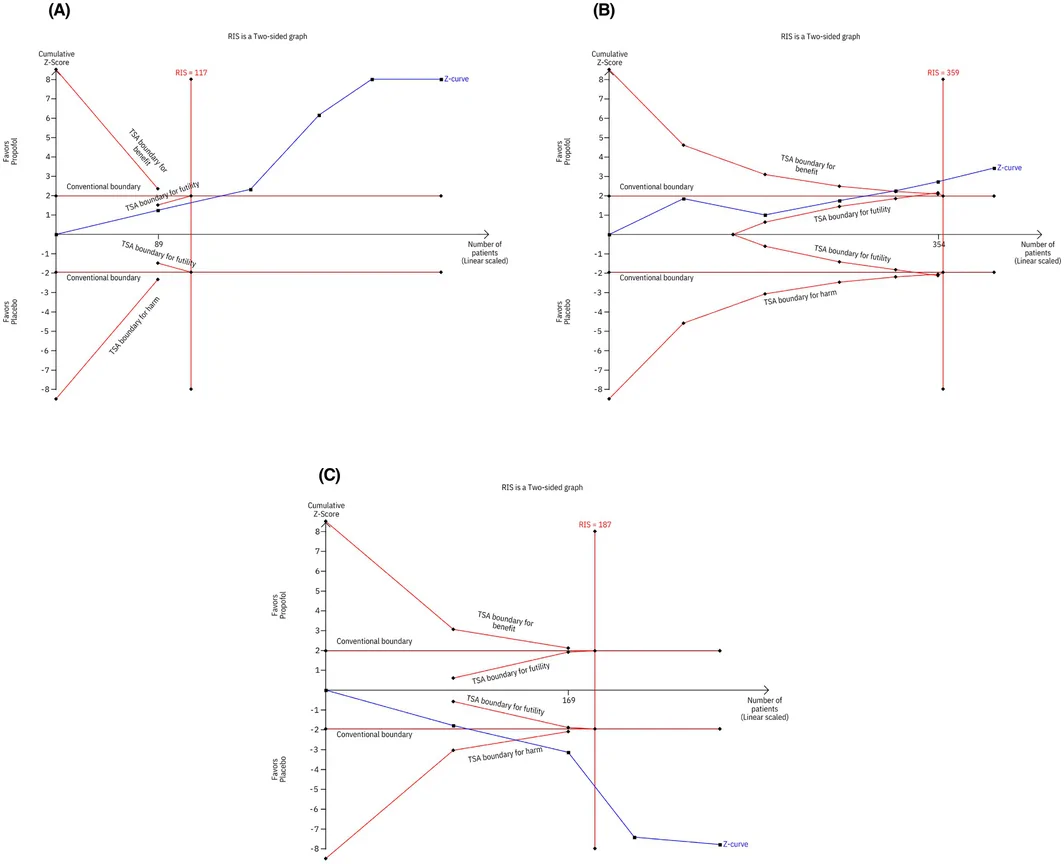
Trial sequential analysis. (A) Trial sequential analysis (TSA) for the severity of emergence agitation. The cumulative Z‐curve (blue line) represents the accumulated evidence. The horizontal red lines indicate conventional significance boundaries, while the sloping red lines represent sequential monitoring boundaries (for benefit, harm, or futility) adjusted for repetitive testing. The vertical red line marks the required information size (RIS = 117), the calculated threshold for an optimal sample size. The cumulative Z‐curve crosses the conventional boundary and explicitly surpasses the RIS threshold, demonstrating that the cumulative sample size is sufficient. (B) Trial sequential analysis (TSA) for the incidence of emergence agitation. The cumulative Z‐curve (blue line) represents the accumulated evidence. The horizontal red lines indicate conventional significance boundaries, while the sloping red lines represent sequential monitoring boundaries (for benefit, harm, or futility) adjusted for repetitive testing. The vertical red line marks the required information size (RIS = 359), the threshold for an optimal sample size. The cumulative Z‐curve crosses both the conventional boundary and the sequential boundary for benefit, and explicitly surpasses the RIS threshold, demonstrating that the cumulative sample size is sufficient and the evidence is conclusive. (C) Trial sequential analysis (TSA) for the time to extubation. The cumulative Z‐curve (blue line) represents the accumulated evidence. The horizontal red lines indicate conventional significance boundaries, while the sloping red lines represent sequential monitoring boundaries (for benefit, harm, or futility) adjusted for repetitive testing. The vertical red line marks the required information size (RIS = 187), the threshold for an optimal sample size. The cumulative Z‐curve crosses both the conventional boundary and the sequential boundary for harm and explicitly surpasses the RIS threshold, demonstrating that the cumulative sample size is sufficient and the evidence is conclusive.

### 3.6 Sensitivity Analyses

Sensitivity analysis for EA severity, as measured by the PAED scale score, showed that the overall effect remained stable across leave‐one‐out analyses, with no individual study substantially influencing the observed heterogeneity (Figure S5A).

The leave‐one‐out analysis for EA incidence yielded consistent results with the primary analysis, although heterogeneity decreased from *I* ^2^ = 54.7%–6.1% after exclusion of Lee [11] (Figure S5B).

For time to extubation, the direction of effect remained unchanged, but statistical significance was lost when Lee [11] or Zhong [18] was omitted. Heterogeneity disappeared after excluding Mahdavi Rad [17] (*I* ^2^ = 0%), indicating that this study was the main source of between‐study heterogeneity (Figure S5C).

For time to recovery, the overall effect remained nonsignificant after omitting Rizk & Samir [16] or Bakhamees [13]; however, exclusion of Mahdavi Rad [17] yielded a significant effect (MD 1.75, 95% CI 0.66–2.84; Figure S5D) and eliminated heterogeneity (*I* ^2^ = 0%).

For PACU length of stay, the overall effect remained nonsignificant regardless of the study omitted, although exclusion of Mahdavi Rad [17] markedly reduced heterogeneity (*I* ^2^ = 15.8%) and slightly shifted the effect estimate toward placebo (Figure S5D). All forest plots from the sensitivity analyses are presented in Figure S5.

### 3.7 Quality Assessment and Certainty of Evidence

The overall risk of bias was low for 7 RCTs and considered to have some concerns for 3 RCTs (Bakhamees [13]; Kaur [29]; Lee [11]; Table S2).

Using the GRADE approach, the overall certainty of evidence was moderate for the incidence of EA and time to extubation; low for time to recovery and PACU length of stay; and very low for EA severity, as measured by the PAED scale score (Table S3). We did not assess publication bias because fewer than 10 studies contributed to each outcome.

## 4 Discussion

Ten RCTs met the inclusion criteria, of which six (414 pediatric patients undergoing ENT surgery) were included in the meta‐analysis comparing propofol 1 mg·kg^−1^ administered during the pre‐extubation period with placebo. All meta‐analyzed studies evaluated a single bolus administered during emergence, between 10 min before surgical completion and the time of extubation. Our findings suggest that pre‐extubation propofol probably reduces the incidence of EA in pediatric ENT surgery, supported by moderate‐certainty evidence, and may lower EA severity measured by PAED scale scores, for which the certainty of evidence was very low. Propofol was also associated with a slightly longer time to extubation, with no difference in overall recovery time or PACU length of stay.

Incidence of EA and severity of EA measured by PAED scores should be interpreted as complementary measures of the same clinical outcome, reflecting whether agitation occurs and its severity when present. Because several studies contributed to both analyses, these findings should not be considered independent sources of evidence. Before the introduction of the PAED scale, emergence agitation was commonly assessed using non‐validated instruments such as Watcha's four‐point scale and Cravero's five‐point scale, which relied on simpler categorical assessments [30]. In contrast, the PAED scale provides greater objectivity, granularity, reliability, and reproducibility by capturing both behavioral features (restlessness and inconsolability) and cognitive features (eye contact, purposeful actions, and awareness of surroundings) of emergence agitation [31]. Consequently, although the certainty of evidence for PAED scores was very low, changes in PAED may represent the most clinically meaningful measure of the impact of propofol on EA, supporting the overall finding that suggests propofol reduces both the occurrence and the clinical expression of EA.

The substantial heterogeneity observed in the analysis of EA severity, as measured by the PAED score, warrants cautious interpretation. Although the PAED scale has demonstrated good overall interrater reliability, item‐level variability has been reported, particularly for cognitive items [22, 32]. However, the observed between‐study heterogeneity is more likely attributable to differences in observer training, assessment timing, and outcome measurement protocols than to limitations of the instrument itself [31]. Indeed, subgroup analysis according to the timing of PAED assessment identified significant subgroup differences, with studies assessing patients on arrival to the PACU demonstrating larger treatment effects. Despite the observed heterogeneity, leave‐one‐out analysis consistently favored propofol, and no individual study was identified as an outlier.

Given that volatile anesthetics, inadequate analgesia, and rapid emergence are established risk factors for EA in this population [2, 7, 33], propofol administered before extubation may mitigate agitation by prolonging neural suppression postoperatively [34, 35]. This effect may involve two main mechanisms: acting as a gamma‐aminobutyric acid receptor agonist, enhancing inhibitory transmission and central sedation [35], and acting as a noncompetitive antagonist of N‐methyl‐D‐aspartate (NMDA) receptors, which may decrease excitatory neurotransmission and dampen circuits linked to anxiety and hyperexcitability [34].

Postoperative pain is a major contributor to emergence agitation following tonsillectomy and adenoidectomy and is an important potential confounder when interpreting preventive strategies [36]. Variability in perioperative analgesic protocols across the included studies may therefore have influenced the observed treatment effects and contributed to between‐study heterogeneity, as pain‐related distress can mimic or exacerbate emergence agitation. In the analysis of EA incidence, sensitivity analysis identified Lee et al. as a potential outlier; excluding this study reduced heterogeneity while preserving the overall treatment effect. The authors of that trial also acknowledged that suboptimal postoperative analgesia may have influenced their findings. Nevertheless, the persistence of benefit after excluding this study suggests that the observed effect of propofol is unlikely to be explained solely by differences in analgesic management. Future trials should incorporate standardized multimodal analgesic protocols to better isolate the independent effect of propofol on EA.

A common concern with any intraoperative sedative adjunct is the risk of delayed recovery or prolonged PACU length of stay, which could increase hospital costs and resource demands. Patients receiving propofol experienced a statistically significant increase in time to extubation. However, this brief delay is unlikely to be clinically meaningful and represents a minimal trade‐off for the observed reduction in EA. This is a key finding, as minimizing EA without prolonging recovery helps prevent unnecessary intensive care unit admissions, improves bed turnover, and optimizes healthcare resources [37, 38].

Several included trials were conducted during an era when thiopental induction, halothane maintenance, and routine nitrous oxide administration were common practices [11, 17]. In contrast, none of the included studies evaluated propofol in conjunction with dexmedetomidine, which is commonly studied for EA prophylaxis [39]. Therefore, the incremental benefit of propofol within modern anesthetic pathways remains uncertain. Given their distinct pharmacologic mechanisms, however, propofol and dexmedetomidine may provide complementary rather than redundant effects, a possibility that warrants investigation in future trials.

This review has several limitations. The absence of standardized definitions of laryngospasm and extubation protocols precluded quantitative synthesis of this outcome. Variability in the assessment tools used to define EA, as well as differences in perioperative analgesic protocols and anesthetic techniques (including the use of thiopental in older studies), may also have contributed to the observed heterogeneity. Consequently, EA severity measured by the PAED score was considered the most informative outcome. Finally, the relatively small sample sizes of the included trials may have inflated treatment effect estimates, and publication bias could not be formally assessed because of the limited number of studies available for each outcome.

Our findings suggest that pre‐extubation propofol probably reduces the incidence of EA in pediatric ENT surgery, supported by moderate‐certainty evidence, and may reduce EA severity, as measured by the PAED score, although the certainty of evidence for this outcome was very low. Propofol was also associated with a slightly longer time to extubation, with no difference in overall recovery time or PACU length of stay. All studies included in the meta‐analysis evaluated a 1 mg·kg^−1^ bolus of propofol administered during emergence, most commonly at or near the end of surgery, compared with saline placebo, and all used awake extubation. Accordingly, the observed reductions in EA were consistently achieved within the context of this regimen. Further studies are needed to determine the optimal timing of propofol administration and its role within contemporary anesthetic protocols incorporating dexmedetomidine and standardized multimodal analgesia.

## Author Contributions

Rafaela Silva made substantial contributions to the conception and design of the study, including development of the PICOTT framework and search strategy; contributed to the acquisition, analysis, and interpretation of data, including resolution of screening disagreements, verification of data extraction, risk of bias assessment, GRADE evaluation, and statistical analyses; participated in drafting and critically revising the manuscript for important intellectual content; approved the final version to be published and serves as corresponding author and guarantor of the work. Raphaela Mendes, MD contributed to the acquisition of data through study screening and data extraction; participated in drafting the manuscript, particularly the Discussion section; and approved the final version to be published. João Mario Borges, MD contributed to the acquisition of data through study screening and data extraction; participated in drafting the manuscript, particularly the Introduction section; and approved the final version to be published. Henrique Pivetta contributed to the acquisition of data, preparation of Table 1, and reference management; participated in drafting and critical revision of the Results section for important intellectual content; and approved the final version to be published. Rafael Miranda contributed to the analysis and interpretation of data, including preparation of Table 1, GRADE assessment, and assistance with statistical analyses; participated in drafting and critical revision of the Results section for important intellectual content; and approved the final version to be published. Isabela Alles contributed to the analysis and interpretation of data through risk of bias assessment and GRADE evaluation; participated in drafting and critical revision of the Results section for important intellectual content; and approved the final version to be published. Vinicius Oliveira contributed to the analysis and interpretation of data through trial sequential analysis; participated in critical revision of the manuscript for important intellectual content; and approved the final version to be published. Steven Staffa contributed to the analysis and interpretation of data through senior statistical review and evaluation of statistical coherence and rigor; participated in critical revision of the manuscript for important intellectual content; and approved the final version to be published. Walid Alrayashi, MD, contributed to the conception and design of the study through refinement of the research idea and methodological supervision; participated in critical revision of the manuscript for important intellectual content; and approved the final version to be published.

## Funding

The authors have nothing to report.

## Ethics Statement

The authors have nothing to report.

## Conflicts of Interest

The authors declare no conflicts of interest.

## Supporting information


**Table S1:** Prisma checklist.
**Figure S1:** Timing of propofol bolus administration subgroup analysis. (A) Subgroup analysis of the timing of propofol bolus administration for the outcome of EA severity, as measured by the PAED scale score. (B) Subgroup analysis of the timing of propofol bolus administration for the outcome of EA incidence.
**Figure S2:** Maintenance agent subgroup analysis. (A) Subgroup analysis of the maintenance agent used for the outcome of EA severity, as measured by the PAED scale score. (B) Subgroup analysis of the maintenance agent used for the outcome of EA incidence.
**Figure S3:** PAED assessment subgroup.
**Figure S4:** Emergence agitation cutoff subgroup analysis.
**Figure S5:** Leave‐one‐out analysis. (A) Leave‐One‐Out analysis for severity of EA, as measured by PAED scale score. (B) Leave‐One‐Out analysis for incidence of emergence agitation. (C) Leave‐One‐Out analysis for time to extubation. (D) Leave‐One‐Out analysis for time to recovery. (E) Leave‐One‐Out analysis for post‐anesthesia unit care length of stay.
**Table S2:** Risk of bias assessment for included studies.
**Table S3:** Summary of findings and certainty of evidence (GRADE).

## Data Availability

The data that support the findings of this study were derived from publicly available studies. No new datasets were generated or analyzed during the current study. All data extracted and analyzed are available within the article and its Appendix S1.
