# Supplementary material for: Pre‐Extubation Low‐Dose Propofol in Pediatric Ear, Nose, and Throat Procedures: A Meta‐Analysis With Trial Sequential Analysis
Source: Paediatr Anaesth. 2026 Aug 12;36(11):1375–86. doi: 10.1002/pan.70277 (PMC13639204; doi:10.1002/pan.70277)
Supplement: Supplementary file 1 [file PAN-36-1375-s001.docx]

**Supplementary Table 1. Prisma checklist**

| **Section and Topic** | **Item #** | **Checklist item** | **Location** |
| --- | --- | --- | --- |
| **TITLE** | | |  |
| Title | 1 | Identify the report as a systematic review. | Page 1 |
| **ABSTRACT** | | |  |
| Abstract | 2 | See the PRISMA 2020 for Abstracts checklist. | Page 5 |
| **INTRODUCTION** | | |  |
| Rationale | 3 | Describe the rationale for the review in the context of existing knowledge. | Page 7 |
| Objectives | 4 | Provide an explicit statement of the objective(s) or question(s) the review addresses. | Page 8 |
| **METHODS** | | |  |
| Eligibility criteria | 5 | Specify the inclusion and exclusion criteria for the review and how studies were grouped for the syntheses. | Page 8 |
| Information sources | 6 | Specify all databases, registers, websites, organisations, reference lists and other sources searched or consulted to identify studies. Specify the date when each source was last searched or consulted. | Page 9 |
| Search strategy | 7 | Present the full search strategies for all databases, registers and websites, including any filters and limits used. | Page 9 and Supplementary material |
| Selection process | 8 | Specify the methods used to decide whether a study met the inclusion criteria of the review, including how many reviewers screened each record and each report retrieved, whether they worked independently, and if applicable, details of automation tools used in the process. | Page 9 |
| Data collection process | 9 | Specify the methods used to collect data from reports, including how many reviewers collected data from each report, whether they worked independently, any processes for obtaining or confirming data from study investigators, and if applicable, details of automation tools used in the process. | Page 9 |
| Data items | 10a | List and define all outcomes for which data were sought. Specify whether all results that were compatible with each outcome domain in each study were sought (e.g. for all measures, time points, analyses), and if not, the methods used to decide which results to collect. | Page 9 |
|  | 10b | List and define all other variables for which data were sought (e.g. participant and intervention characteristics, funding sources). Describe any assumptions made about any missing or unclear information. | Page 9 |
| Study risk of bias assessment | 11 | Specify the methods used to assess risk of bias in the included studies, including details of the tool(s) used, how many reviewers assessed each study and whether they worked independently, and if applicable, details of automation tools used in the process. | Page 10 |
| Effect measures | 12 | Specify for each outcome the effect measure(s) (e.g. risk ratio, mean difference) used in the synthesis or presentation of results. | Page 10 |
| Synthesis methods | 13a | Describe the processes used to decide which studies were eligible for each synthesis (e.g. tabulating the study intervention characteristics and comparing against the planned groups for each synthesis (item #5)). | Page 10 |
|  | 13b | Describe any methods required to prepare the data for presentation or synthesis, such as handling of missing summary statistics, or data conversions. | Page 10 |
|  | 13c | Describe any methods used to tabulate or visually display results of individual studies and syntheses. | Page 10 |
|  | 13d | Describe any methods used to synthesize results and provide a rationale for the choice(s). If meta-analysis was performed, describe the model(s), method(s) to identify the presence and extent of statistical heterogeneity, and software package(s) used. | Page 11 |
|  | 13e | Describe any methods used to explore possible causes of heterogeneity among study results (e.g. subgroup analysis, meta-regression). | Page 11 |
|  | 13f | Describe any sensitivity analyses conducted to assess robustness of the synthesized results. | Page 11 |
| Reporting bias assessment | 14 | Describe any methods used to assess risk of bias due to missing results in a synthesis (arising from reporting biases). | Page 9 |
| Certainty assessment | 15 | Describe any methods used to assess certainty (or confidence) in the body of evidence for an outcome. | Page 9 |
| **RESULTS** | | |  |
| Study selection | 16a | Describe the results of the search and selection process, from the number of records identified in the search to the number of studies included in the review, ideally using a flow diagram. | Page 11 and Figure 1 |
|  | 16b | Cite studies that might appear to meet the inclusion criteria, but which were excluded, and explain why they were excluded. | Page 11 |
| Study characteristics | 17 | Cite each included study and present its characteristics. | Page 12 |
| Risk of bias in studies | 18 | Present assessments of risk of bias for each included study. | Page 16 |
| Results of individual studies | 19 | For all outcomes, present, for each study: (a) summary statistics for each group (where appropriate) and (b) an effect estimate and its precision (e.g. confidence/credible interval), ideally using structured tables or plots. | Page 13 |
| Results of syntheses | 20a | For each synthesis, briefly summarise the characteristics and risk of bias among contributing studies. | Page 13 |
|  | 20b | Present results of all statistical syntheses conducted. If meta-analysis was done, present for each the summary estimate and its precision (e.g. confidence/credible interval) and measures of statistical heterogeneity. If comparing groups, describe the direction of the effect. | 13 |
|  | 20c | Present results of all investigations of possible causes of heterogeneity among study results. | Page 14 |
|  | 20d | Present results of all sensitivity analyses conducted to assess the robustness of the synthesized results. | Page 14 |
| Reporting biases | 21 | Present assessments of risk of bias due to missing results (arising from reporting biases) for each synthesis assessed. | Page 17 |
| Certainty of evidence | 22 | Present assessments of certainty (or confidence) in the body of evidence for each outcome assessed. | Page 17 |
| **DISCUSSION** | | |  |
| Discussion | 23a | Provide a general interpretation of the results in the context of other evidence. | Page 17 |
|  | 23b | Discuss any limitations of the evidence included in the review. | Page 20 |
|  | 23c | Discuss any limitations of the review processes used. | Page 20 |
|  | 23d | Discuss implications of the results for practice, policy, and future research. | Page 20 |
| **OTHER INFORMATION** | | |  |
| Registration and protocol | 24a | Provide registration information for the review, including register name and registration number, or state that the review was not registered. | Page 9 |
|  | 24b | Indicate where the review protocol can be accessed, or state that a protocol was not prepared. | Page 9 |
|  | 24c | Describe and explain any amendments to information provided at registration or in the protocol. | Page 9 |
| Support | 25 | Describe sources of financial or non-financial support for the review, and the role of the funders or sponsors in the review. | Page 2 |
| Competing interests | 26 | Declare any competing interests of review authors. | Page 2 |
| Availability of data, code and other materials | 27 | Report which of the following are publicly available and where they can be found: template data collection forms; data extracted from included studies; data used for all analyses; analytic code; any other materials used in the review. | Supplementary material |

**=**

**Full search strategy.**

- PubMed

(pediatric OR paediatric OR children OR child OR kid OR infant OR toddler OR adolescent OR neonate) AND (tonsillectomy OR adenoidectomy OR "tonsil removal" OR "adenoid removal" OR adenotonsillectomy OR "ENT surgery" OR otolaryngologic OR "head and neck surgery" OR "ear nose throat" OR pharyngeal OR nasopharyngeal OR oropharyngeal) AND (propofol OR Diprivan)

- Embase

(pediatric* OR paediatric* OR child* OR kid* OR infant* OR toddler* OR adolescent* OR neonate* OR 'child'/exp OR 'infant'/exp OR 'adolescent'/exp OR 'neonate'/exp) AND (tonsillectomy OR adenoidectomy OR adenotonsillectomy OR "tonsil removal" OR "adenoid removal" OR "ENT surgery" OR "ear nose throat surgery" OR otolaryngologic OR "head and neck surgery" OR pharyngeal OR nasopharyngeal OR oropharyngeal OR 'tonsillectomy'/exp OR 'adenoidectomy'/exp OR 'otolaryngology'/exp OR 'head and neck surgery'/exp) AND (propofol OR Diprivan OR 'propofol'/exp)

- Cochrane Library

(pediatric OR paediatric OR children OR child OR kid OR infant OR toddler OR adolescent OR neonate) AND (tonsillectomy OR adenoidectomy OR "tonsil removal" OR "adenoid removal" OR adenotonsillectomy OR "ENT surgery" OR otolaryngologic OR "head and neck surgery" OR "ear nose throat" OR pharyngeal OR nasopharyngeal OR oropharyngeal) AND (propofol OR Diprivan)

**Supplementary Figure 1. Timing of propofol bolus administration subgroup analysis**

A) **Subgroup analysis of the timing of propofol bolus administration for the outcome of EA severity, as measured by the PAED scale score.**

CI: confidence interval; MD: mean difference.

B) **Subgroup analysis of the timing of propofol bolus administration for the outcome of EA incidence.**

CI: confidence interval; RR: risk ratio.

**Supplementary Figure 2. Maintenance agent subgroup analysis**

A) **Subgroup analysis of the maintenance agent used for the outcome of EA severity, as measured by the PAED scale score.**

CI: confidence interval; MD: mean difference.

**B) Subgroup analysis of the maintenance agent used for the outcome of EA incidence.**

CI: confidence interval; RR: risk ratio.

**Supplementary Figure 3. PAED assessment subgroup**

CI: confidence interval; MD: mean difference.

**Supplementary Figure 4. Emergence agitation cutoff subgroup analysis**

CI: confidence interval; RR: risk ratio.

**Supplementary Figure 5. Leave-one-out analysis**

A) **Leave-One-Out analysis for severity of EA, as measured by PAED scale score.**

The results remained consistent despite the removal of individual studies. CI: confidence interval; MD: mean difference.

B) **Leave-One-Out analysis for incidence of emergence agitation.**

The results remained consistent despite the removal of individual studies. CI: confidence interval; RR: risk ratio.

C) **Leave-One-Out analysis for time to extubation**

No heterogeneity when omitting Rad 2019 from the analysis. CI: confidence interval; RR: risk ratio.

D) **Leave-One-Out analysis for time to recovery.**

No heterogeneity when omitting Rad 2019 from the analysis. CI: confidence interval; RR: risk ratio.

. CI: confidence interval; RR: MD: mean difference

E) **Leave-One-Out analysis for post-anesthesia unit care length of stay.**

The results remained inconclusive despite the removal of individual studies. CI: confidence interval; MD: mean difference

**Supplementary Table 2. Risk of bias assessment for included studies.**

| **Study** | **Randomization process** | **Deviations from intended interventions** | **Due to missing outcome data** | **Measurement of the outcomes** | **Selection of the reported result** | **Overall risk of bias** |
| --- | --- | --- | --- | --- | --- | --- |
| Ali, 2013 | Low | Low | Low | Low | Low | Low |
| Bakhamees, 2009 | Some concerns | Low | Low | Low | Low | Some concerns |
| Batra, 2005 | Low | Low | Low | Low | Low | Low |
| Hosseini, 2021 | Low | Low | Low | Low | Low | Low |
| Iqbal, 2017 | Low | Low | Low | Low | Low | Low |
| Kaur, 2006 | Some concerns | Low | Low | Some concerns | Low | Some concerns |
| Lee, 2010 | Low | Low | Low | Some concerns | Low | Some concerns |
| Rad, 2019 | Low | Low | Low | Low | Low | Low |
| Rizk, 2013 | Low | Low | Low | Low | Low | Low |
| Zhong, 2018 | Low | Low | Low | Low | Low | Low |

**Supplementary Table 3. Summary of findings and certainty of evidence (GRADE).**
